# Supplementation with Synbiotics and/or Branched Chain Amino Acids in Hepatic Encephalopathy: A Pilot Randomised Placebo-Controlled Clinical Study

**DOI:** 10.3390/nu11081810

**Published:** 2019-08-06

**Authors:** Helen Vidot, Erin Cvejic, Liam J. Finegan, E. Arthur Shores, David G. Bowen, Simone I. Strasser, Geoffrey W. McCaughan, Sharon Carey, Margaret Allman-Farinelli, Nicholas A. Shackel

**Affiliations:** 1 Department of Nutrition and Dietetics, Royal Prince Alfred Hospital, Camperdown, NSW 2050, Australia; 2 Liver Injury and Cancer Centre, Centenary Research Institute, The University of Sydney, Sydney, NSW 2006, Australia; 3 School of Public Health, The University of Sydney, Sydney, NSW 2006, Australia; 4 School of Business, The University of Sydney, Sydney, NSW 2006, Australia; 5 Department of Psychology, Macquarie University, Sydney, NSW 2109, Australia; 6 Sydney Medical School, The University of Sydney, Sydney, NSW 2006 Australia; 7 A.W. Morrow Gastroenterology and Liver Centre, Royal Prince Alfred Hospital, Camperdown, NSW 2050, Australia; 8 School of Life and Environmental Sciences Charles Perkins Centre, The University of Sydney, Sydney, NSW 2006, Australia; 9 Department of Medicine, University of New South Wales, Sydney, NSW 2052, Australia

**Keywords:** hepatic encephalopathy, synbiotics, branched chain amino acids, trail making test, inhibitory control test, cognition

## Abstract

Introduction: Hepatic encephalopathy (HE) is common in patients with cirrhosis and is characterised by reduced hepatic ammonia clearance. This is accompanied by alterations in gut bacteria that may be ameliorated with synbiotics (pro- and prebiotics). Branched chain amino acids (BCAAs) are thought to have a role in the detoxification of ammonia. We investigated the effects of the administration of synbiotics and/or BCAAs in treating HE. Methods: Participants with overt HE were randomised in a blinded placebo-controlled study to receive synbiotics, BCAAs, or a combination of BCAAs and Synbiotics. Relevant biochemical and nutritional data and depression and anxiety scores (DASS-21) were collected at entry, 4 weeks, and on completion, at 8 weeks. The Trail Making Test (TMT) and Inhibitory Control Test (ICT) were used to assess cognitive function in patients withHE. Results were analysed using linear mixed effects regression analyses. Results: Sixty-one participants were enrolled and 49 who returned for at least 1 follow-up review were included in the intention to treat analysis. The mean age was 55.8 ± 6.1 years and 86% were males. Despite evidence of a placebo effect, there was significant improvement in TMT B and ICT weighted lures in participants who received combined synbiotics/BCAAs treatment compared to placebo at study completion (*p* ≤ 0.05). Cognitive improvement occurred without a significant change in ammonia levels. Conclusion: To our knowledge, this is the first study reporting that combined synbiotics and BCAAs improve HE, and that may be beneficial in the management of HE. A larger study is needed to confirm these results.

## 1. Introduction

Hepatic encephalopathy (HE) is a term that describes a wide range of nonspecific, potentially reversible, neurophysiological changes associated with hepatic insufficiency in individuals with chronic liver disease [1]. Its occurrence relates to the progression and severity of hepatic cirrhosis and portosystemic shunting [1]. HE may be minimal, episodic, or persistent [2]. Episodic HE may be spontaneous without an identifiable precipitant, or result from a number of clinical changes which include gastrointestinal bleeding, infection, constipation, hypoglycaemia, or hyponatremia [3] Persistent HE is associated with chronic alterations in cognitive and motor function, which may range from mild or severe [3]. 

The nomenclature and classification of HE have been refined and current schema classify HE as either covert or overt HE [3]. Covert HE is estimated to occur in 30–85% of patients with cirrhosis and is characterised by mild cognitive abnormalities [4]. Overt HE, which is characterised by more severely impaired mental status and neuromotor function, is associated with reduced quality of life, increased somnolence, increased frequency of hospital admissions, and increased mortality [4]. It occurs in 30–50% of patients with decompensated cirrhosis [4]. Both covert and overt HE present a significant burden for caregivers and health care systems [5]. 

Overt HE occurs in 30–40% of patients with cirrhosis [3]. Persistent or repeated episodes of overt HE may result in cumulative deficits in working memory, response inhibition, and learning [6], which may not be fully resolved following liver transplantation [7]. A number of neuropsychological tests have been developed to detect deficits that characterise hepatic encephalopathy [2]. They include, but are not limited to, the Inhibitory Control Test (ICT) [8] and the Trail Making Test (TMT) [9]. 

Ammonia has been considered both a factor in the development of HE and a marker of HE, but rising serum ammonia concentrations do not correlate with the symptoms of HE [10]. Ammonia homeostasis primarily involves the liver [10], but ammonia is also produced by enterocytes and colonic bacteria [11]. Elevated levels of circulating ammonia induce neutrophil dysfunction, resulting in a reduced inflammatory response, increased oxidative stress [12], altered neurotransmission, increased brain oedema, and astrocyte swelling [13]. It is generally accepted that the development of HE is, in part, a consequence of a systemic inflammatory state with increased circulating levels of ammonia arising from dysbiosis and alterations in gut permeability [12]

Since the late 1980s, when the concept of the gut–liver–brain axis was first introduced, there has been a growing awareness and acceptance of the interplay between the gut, the composition of the gut bacteria, the liver, and the brain [14]. The gut epithelium is a highly regulated selective natural barrier [15]. There is a causal relationship between alterations in bile acid production, reduced gut bacterial diversity and richness, luminal dysbiosis, cirrhosis severity, and cognitive performance [12,13,16]. 

A functional dysbiosis has been identified in patients with cirrhosis, which was associated with increased markers of systemic inflammation and HE [17]. Supplementation with probiotics, prebiotics, or both may promote the growth and diversity of beneficial bacteria and ultimately may result in an improvement in brain function and cognition [12]. 

A Cochrane review of 21 trials of probiotic supplementation in patients with hepatic encephalopathy concluded that probiotic use was associated with an improvement in the symptoms of HE, a delay in the development of overt HE, and an improvement in the quality of life, compared to placebo or no intervention. Importantly, there were no reports of septicaemia associated with probiotic use [18].

A consequence of the nutritional changes accompanying chronic liver disease [19] is a reduction in circulating levels of branched chain amino acids (BCAA: leucine, isoleucine, and valine) and an increase in circulating levels of the aromatic amino acids (phenylalanine, tyrosine, and tryptophan) [20]. BCAAs improve glucose metabolism, promote protein synthesis, inhibit proteolysis in skeletal muscle, and are involved in ammonia detoxification via the glutamate/glutamine pathway which requires ammonia [21]. A recent Cochrane review supports the beneficial use of oral BCAA supplementation in reducing HE [22].

The principle aim of this pilot study was to investigate the effect of oral supplementation with synbiotics and/or BCAAs on HE in patients who were receiving ongoing treatment with lactulose. Additional outcomes included the effect of supplementation on physiological markers of infection, macronutrient intakes, depression, anxiety and stress, and body composition. 

## 2. Materials and Methods 

### 2.1. Ethics and Consent

Signed, informed consent was obtained from all participants prior to enrolment and all research was conducted in accordance the Declaration of Helsinki and with the approval of the Sydney Local Health District Human Research Ethics Committee (RPAH zone: X09-006 and HREC/09/RPAH/5). The investigation was registered with the Australian New Zealand Clinical Trials register: ACTRN12610001021066. 

### 2.2. Participant Selection

Adult patients with hepatic cirrhosis and a history of HE (West Haven 1,2) [23] who attended a liver clinic were invited to participate in a study which investigated the effect of supplementation with synbiotics, BCAAs, combined synbiotics and BCAAs, or placebo. Participants were required to be on daily lactulose therapy, abstinent from alcohol and intravenous drug use for at least 3 months prior to entry to the study, and, if on methadone, were required to be dose-stable for a minimum of 3 months prior to study entry. Individuals with coeliac disease or a history of gluten sensitivity were excluded from the study due to the composition of the synbiotics. Potential participants were excluded if they were currently using a probiotic or if they were taking rifaximin, an antibiotic now widely used in the management of HE [24], or if random blood glucose levels were ≥15mMol/L. At the time of the study design and initiation, rifaximin was not available in Australia for the treatment of HE and it only became available during the period of study recruitment. At the time of recruitment to the study, availability of microbiota analysis of stool samples and measurement of intestinal epithelial permeability was not available. 

### 2.3. Psychometric Testing Methods

Prior to randomisation, hepatic encephalopathy was confirmed by the treating hepatologist and documented regular lactulose use. HE was further confirmed by psychometric testing which included both the computerised ICT [8] and pencil-paper TMT [9]. The ICT is a computerised neuropsychological test of working memory, attention, processing speed, and inhibition or self-control [8]. The TMT is a simple pencil-paper test which is widely used to assess cognitive processes including attention, visual search and scanning, sequencing and shifting, graphomotor speed, abstraction, flexibility, ability to execute and modify a plan of action, and the ability to maintain two trains of thought simultaneously [9,25,26]. 

The results of the ICT were expressed as number of targets, target accuracy, number of non-inhibited responses or lures, and weighted lures [9,27]. TMT results were expressed in seconds and compared with an Australian normative sample [28].

Inhibition is one of the most frequently used cognitive functions and can be learned, trained, and improved [29]. Disinhibition on a cognitive level will make it difficult to inhibit distracting stimuli [29]. Testing for attention is as important as testing for inhibition to diagnose cognitive dysfunction in patients with cirrhosis [2]. 

As the TMT and ICT responses were major measures of cognition and executive functioning, the primary investigator explained both the TMT and the ICT in great detail prior to participants commencing the tests. Some participants required more than 1 practice run to confirm that they understood the tasks. 

### 2.4. Participant Allocation

As this was a pilot study to obtain preliminary data to justify further definitive investigations of these treatments, a power calculation was not considered. The study aimed to recruit 80 participants with even allocation (*n* = 20) to each intervention.

**Figure 1:**
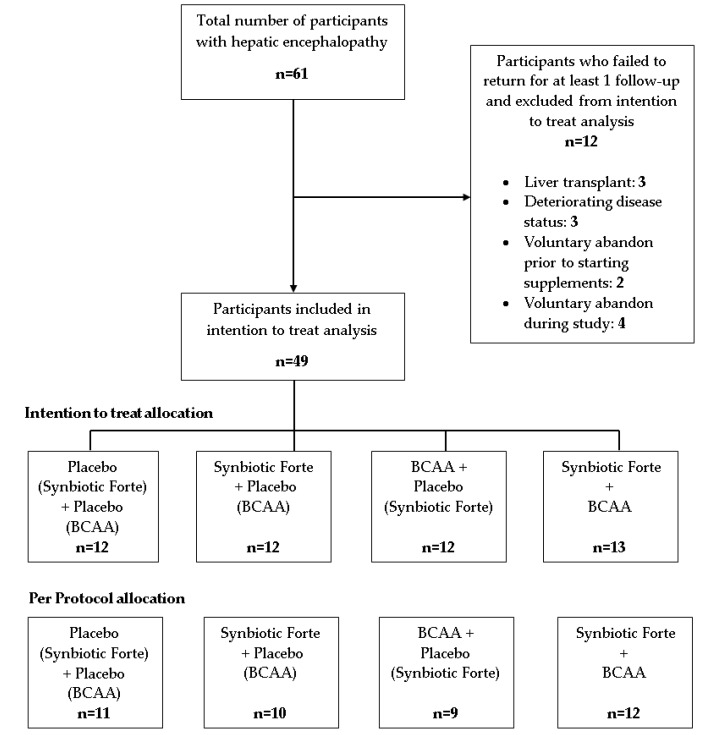
Study Design and Patient Allocation. Figure 1 outlines the allocation of eligible participants to the 4 treatment arms. Twelve participants did not return for at least 1 follow-up assessment and were not be included in the intention to treat analysis for the reasons indicated. BCAA, branched chain amino acids. On recruitment, participants were invited to randomly select a sealed, opaque envelope from a box which contained all treatments and were subsequently allocated to the randomised intervention. The synbiotic supplement or placebo was taken in the morning and the BCAA supplement or placebo was taken at night before bed. All participants were required to use a standard high protein, high calorie sip supplement which provided 18 g protein and 250 kcals in addition to their usual diet.

Following informed, signed consent, participants were randomly allocated to one of four arms [30] to receive either: Synbiotic 2000 Forte™ and branched chain amino acids (BCAA), Synbiotic 2000 Forte™ and placebo for BCAA, placebo for Synbiotic Forte and BCAA or placebo for Synbiotic Forte and placebo for BCAA (Figure 1). 

### 2.5. Interventions Assessed

Each 10 g sachet of Synbiotic 2000 Forte™ contained a mixture of 4 probiotics (Lactobacillus paracasei ssp paracasei 10 × 10¹¹, Lactobacillus plantarum 10 × 10¹¹, Leuconostoc mesenteroides 10 × 10¹¹, and Pediococcus pentosaceus10 × 10¹¹) and 4 fibres (2.5 g oat bran, 2.5 g pectin, 2.5 g resistant starch, and 2.5 g inulin). The placebo for synbiotics contained 10 g crystalline starch. 

The BCAA supplement used was Hepatamine^®^ and each sachet provided 2.325 g leucine, 1.875 g isoleucine, 1.8 g valine, and 180 kcals. The placebo for Hepatamine was a mixture of an orange flavoured powdered drink base and glucose which was isocaloric and provided 180 kcals. 

Participants were blinded to synbiotics but due to packaging and stability of the BCAA supplement, they could not be blinded to the BCAA supplement and its placebo. Investigators were blinded to both supplements and their placebo at all times. In order to maintain investigator blinding, the supplements and their placebo were distributed by a nurse not associated with the study. All returns were handled and recorded by an independent party.

### 2.6. Additional Data

Standard biochemical indicators of liver function, neutrophil:lymphocyte ratio (NLR), measures of nutritional status including subjective global assessment (SGA) [31], hand grip strength and mid-arm muscle circumference [18], 3-day food intake food diaries, all medications, either physician prescribed or self-prescribed, and depression and anxiety scores [32] were collected at baseline, at 4 weeks and at 8 weeks on completion of the study. Disease severity was assessed by Model for End-stage Liver Disease (MELD) and Child Pugh (CP) scores [33]. FoodWorks Professional^®^ [34] was used to analyse nutrient intakes from the 3-day food diaries recorded by participants.

### 2.7. Mood and Cognitive Assessment of Participants

The Depression and Anxiety Stress Scale-21 (DASS-21), a self-administered shortened form of the 42-question DASS was used to assess participants’ negative emotional states [32]. DASS-21 is more appropriate for clients with limited concentration and aims to separate the core symptoms of depression, anxiety, and stress and is responsive to alterations in clinical status [35].

It is well recognised that there is a learning effect with repetitive administration of psychometric tests in the normal population and that individuals with impaired brain function have a smaller learning effect [36]. The ICT and TMT B are tools that are used clinically to assess HE [8,11]. They evaluate sustained attention and the ability to inhibit responses to potentially relevant stimuli during a stressful working memory updating condition [8,9].

The TMT is a pencil-paper test which measures graphomotor speed, mental flexibility, visual scanning, sequencing, and attention, and results are expressed as time taken to perform the task. TMT A requires participants to join 25 numbers scattered across the page in the correct order and is a simpler test than TMT B. TMT A serves as a baseline for the more complex TMT B which requires greater cognitive ability, wherein participants are required to alternate between numerical and alphabetical systems [28]. 

The ICT is a fast-paced task that assesses working memory, learning capacity, and response inhibition [6] used to identify minimal hepatic encephalopathy [8]. A sequence of letters is presented on a computer screen and subjects are instructed to respond to alternating patterns of the letters X and Y (targets) [8]. Non-alternating presentations of the letters X and Y are randomly planted in the sequence of letters (lures) [8]. There are 212 targets and 40 lures [8]. Patients with cirrhosis have a higher incorrect lure response than normal healthy controls [27]. ICT is influenced by factors such as age, level of education, and familiarity with computers. ICT becomes more relevant if lure outcomes, which measure inhibition ability, are adjusted by target accuracy, a measure of attention ability [27]. The sensitivity and specificity of the weighted lure response in an Italian study was maintained after adjusting for level of education [27]. As there was a wide variation in the level of education attained by the individual participants within our group, the weighted lure response was calculated to identify changes in HE. 

### 2.8. Statistical Analysis

Demographical and clinical characteristics were compared between intervention groups using chi-square tests for categorical data, and one-way analysis of variance for continuous variables. Group performance at baseline on the TMT A and TMT B was compared to normative data [28] using independent sample t-tests (with a Welch correction to the degrees of freedom due to unequal variance). Primary and secondary outcome data were analysed using linear mixed effects regression models, with intervention, study time point (baseline, 4 weeks, 8 weeks), and their interaction included as fixed effects and participant ID treated as a random effect. Analysis was performed as intention-to-treat (ITT). However, it was recognised that participants may forget to take the prescribed treatment at least once weekly, and as such, an “as-treated” or per protocol (PP) analysis using 75% compliance was performed and reported [37]. Stata 15.1 (StataCorp, College Station, Texas, USA) was used to conduct analyses. Given the exploratory nature of the work reported, two-tailed *p*-values less than 0.05 were considered statistically significant; no corrections were made for multiple comparisons.

## 3. Results

Sixty-one participants with a history of minimal (MHE), taking 63 ± 5.7 mL lactulose/day, were recruited into the study. The intention-to-treat (ITT) analysis included all participants who returned for at least one follow-up visit at 4 weeks and/or 8 weeks (*n* = 49). The PP analysis included participants who had a ≥75% compliance (*n* = 43). The study was abandoned prior to the completion of recruitment due to the introduction and widespread use of rifaximin as a primary treatment for HE during the study [23]. 

There were no adverse effects reported with either the BCAAs, synbiotics, or the combined synbiotic and BCAA treatment during this study.

Participants who failed to return for at least one follow-up visit did so for a variety of reasons including liver transplantation (*n* = 3), deteriorating disease status (*n* = 3), voluntary abandon prior to starting intervention (*n* = 2), or voluntary abandon during the study (*n* = 4) (Figure 1). Two participants who had significantly decompensated cirrhosis (CP C) prior to entry withdrew during the study due to disease progression.

Detailed baseline data are set out in Table 1. 

No significant differences between randomised groups were observed on any measure at baseline. ANOVA analyses of both ITT and PP did not identify a significant change in lactulose doses during the study period across the cohort and there were no significant alterations in lactulose doses within intervention groups (*p* = 0.5). Eighty-six percent of participants were males and the average age of participants at enrolment was 55.8 ± 6.1 years. The most common disease aetiology was viral hepatitis (54%), followed by alcohol-related liver disease, and non-alcoholic steatohepatitis. Mean serum ammonia concentration at enrolment was 80.9 ± 47.7 μmol/L which was more than twice the upper limit of the reference range (32 µmol/L). On enrolment into the study MELD, CP score, and NLR were elevated. There were no significant differences in MELD and CP scores across groups at each time interval indicating disease stability through the study period including in the placebo group. Dietary protein intake was 1.3 g/kg/day and was constant across all treatment groups for the duration of the study. 

There was a high prevalence of malnutrition within the study population demonstrated by the SGA results [30] which identified 71% of participants (*n* = 34) as malnourished at enrolment. Mean baseline mid-arm muscle circumference was <35th percentile and dominant hand grip strength was 61% of predicted result. Body composition was unchanged throughout the study. Average performance on both the TMT A and TMT B at baseline was significantly impaired (*p* < 0.001) in comparison to the normative sample [27]. 

There was a trend towards reduced ammonia levels at four weeks in the BCAA alone treatment group compared to placebo (ITT: *p* = 0.07; PP: *p* = 0.08) but there was no difference in ammonia levels in the BCAA alone treatment group at eight weeks compared to placebo (ITT: *p* = 0.10; PP: *p* = 0.12). Further, there were no differences in ammonia for the synbiotic alone group or the combined synbiotic and BCAA treatment group over time in both the ITT and PP groups.

### Psychometric Response to Interventions

TMT performance is set out in Table 2. 

**Figure 2:**
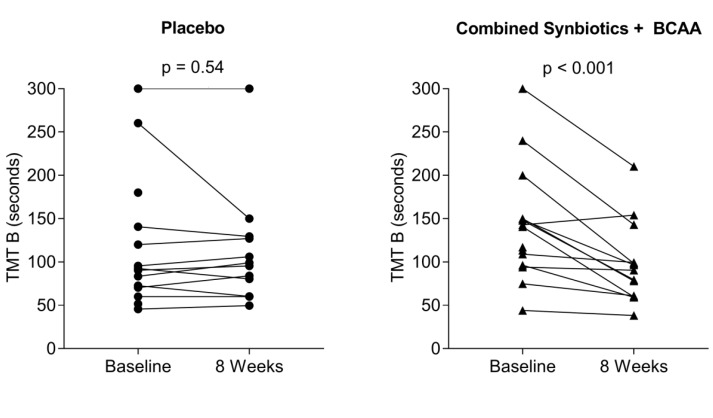
TMT-B performance at Baseline and 8 weeks. TMT B performance at baseline and eight weeks in the placebo treatment group (left panel) and combined synbiotics and BCAA treatment group (right panel). Results demonstrated a significant improvement in TMT B performance from baseline to eight weeks (*p* = <0.001) in the combined synbiotics and BCAA group compared to the responses in the placebo group, who showed no improvement in performance between baseline and eight weeks (*p* = 0.54).

TMT A showed a small but significant improvement over time (ITT: *p* = 0.006; PP: *p* = 0.009) in the placebo group, likely reflecting a practice effect. The intervention by study time interaction provided little statistical evidence that performance differed over time across intervention groups (ITT: *p* = 0.07; PP: *p* = 0.19). For the more complex TMT B, there was no statistical evidence of performance improvement over time in the placebo group (ITT: *p* = 0.09; PP: *p* = 0.07). However, there was statistical evidence that the changes over time differed across intervention groups (intervention by time interaction, ITT: *p* = 0.002; PP: *p* = 0.01). Compared with the placebo group, the combined synbiotic and BCAA group showed significantly greater improvements at 8 weeks relative to the baseline placebo group (ITT: *p* = 0.018; PP: 0.017). The intervention by time interaction in the placebo group and in the combined synbiotic and BCAA group are demonstrated in Figure 2. Relative to changes observed in the placebo group, there were no greater differences in TMT B performance in the synbiotic or BCAA groups compared to baseline. 

ICT responses are outlined in Table 3.

There were no differences in the number of correct target responses across time (ITT: *p* = 0.11; PP: 0.15) and no evidence of an interaction between intervention and time (ITT: *p* = 0.37; PP: *p* = 0.37) was observed. Similarly, target accuracy did not improve over time (ITT: *p* = 0.11; PP: *p* = 0.16), nor were differences seen across interventions over time. However, the decrease in performance from baseline to four weeks was much larger for those in the placebo group compared to the synbiotic group (ITT: *p* = 0.007; PP: *p* = 0.008). 

For lure responses, significantly fewer lures were responded to at both four weeks (ITT: *p* = 0.049; PP: *p* = 0.023) and eight weeks (ITT: *p* = 0.011; PP: *p* = 0.007) compared to baseline in the placebo group, likely reflecting a learning effect due to task repetition. However, there was no evidence that changes in performance over time differed across the intervention groups (ITT: *p* = 0.52; PP: *p* = 0.57). 

**Figure 3:**
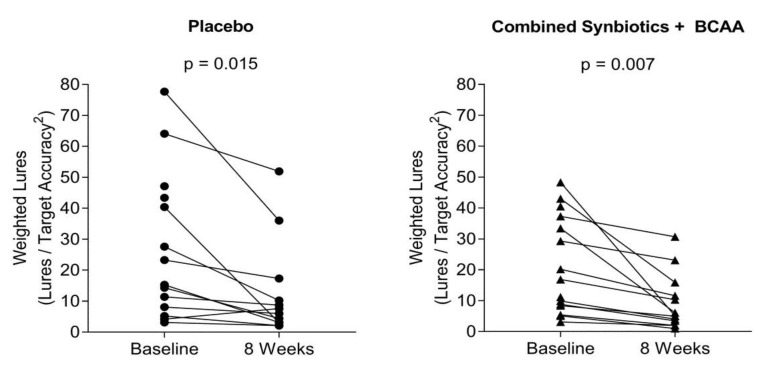
Lure performance at baseline and 8-weeks. Weighted lure performance at baseline and eight weeks in the placebo group (left panel) and combined synbiotics and BCAA treatment group (right panel). Results demonstrated a significant improvement in weighted lure performance from baseline in the combined synbiotics and BCAA group (*p* = 0.007) compared to the placebo group (*p* = 0.015).

Similarly, a significant reduction in weighted lures was observed at both four weeks (ITT: *p* = 0.049; PP: *p* = 0.023) and eight weeks (ITT: *p* = 0.049; PP: *p* = 0.023) for all groups compared to baseline in the placebo group. The interaction between intervention and time was statistically significant (ITT: *p* = 0.015; PP: *p* = 0.05). For the ITT analysis, this interaction was driven by a greater change from baseline in the synbiotic and BCAA alone groups at both four weeks (synbiotic: *p* = 0.015; BCAA: *p* = 0.026) and eight weeks (synbiotic: *p* = 0.037; BCAA: *p* = 0.01) compared to the changes observed in the placebo group (Figure 3). In contrast, the interaction from the PP analyses was likely driven by a greater increase in weighted lure responses between baseline and four weeks follow-up in the combined synbiotics and BCAA group relative to the changes over time in the placebo group (*p* = 0.029). 

The ITT and PP responses to the DASS-21 are outlined in Table 4. There were no significant changes over time in levels of depression and stress as assessed by the DASS-21 across the treatment groups compared to placebo in either the ITT or PP analysis. However, there was weak statistical evidence of a greater decrease in anxiety at eight weeks relative to baseline in the combined synbiotics and BCAA group compared to the placebo group in the ITT analysis (*p* = 0.06). Similarly, in the PP analysis, weak statistical evidence of a greater decrease in anxiety in the combined synbiotics and BCAA group between baseline and four weeks compared to placebo (*p* = 0.055) was observed, but the contrast achieved statistical significance at eight weeks (*p* = 0.035).

## 4. Discussion

The principal findings of this study were an improvement in cognitive performance with combined synbiotic and BCAA treatment in individuals with HE. Cognitive improvement at conclusion of the study was not associated with a significant reduction in serum ammonia. Consistent with other research, this randomised trial of HE treatment had a number of recognised limitations including a marked placebo effect, patient heterogeneity, poor compliance [38], and widespread adoption of other treatment options that lead to difficultly recruiting patients [26]. 

All patients were shown to be encephalopathic throughout the study and due to the episodic nature of HE [11], individual participants were functioning variably at the time of testing. However, there was evidence of improvement in TMT B performance and the ICT weighted lures only in participants who received the combined symbiotic and BCAAs treatment, indicating a reduction in the level of HE in this group. 

MELD, CP, and NLR are markers of disease severity and the results indicate significant decompensated cirrhosis in all participants at levels associated with overall increased morbidity and reduced survival [39]. 

Elevated serum ammonia concentrations are associated with the development of HE and were elevated in all patients, which is consistent with other studies [10]. There was no significant change in serum ammonia concentrations between the placebo and treatment groups after eight weeks, which may reflect the results of previous studies showing ammonia concentration was not associated with the severity of HE [10]. However, the trend towards lower serum ammonia concentrations between the placebo group and the BCAA treatment group at four weeks (*p* = 0.07) did not persist at eight weeks, which may be a consequence of the small number of subjects enrolled in the study.

Our study is consistent with previous studies which demonstrated significant learning effects in participants who completed TMT A [36] that were not replicated in TMT B testing [36]. The TMT A learning effect may last at least until six weeks [36]. Our results failed to demonstrate any significant differences in the TMT A results between placebo and treatment groups which may simply reflect a learning effect across the groups on TMT A, or inadequate sample numbers to detect small effect sizes. Supplementation with synbiotics alone did not appear to improve levels of HE as demonstrated by poorer TMT B performance at four weeks and eight weeks. However, those who received the combined synbiotics and BCAA treatment had significantly reduced TMT B times at eight weeks (*p* = 0.001) indicating an improvement in HE. It appears from these results that TMT B is a sensitive measure capable of detecting differences in cognition in patients with HE.

The results also indicate that ICT weighted lures may be more sensitive to alterations in HE than lure response or target response, which is consistent with the results of a previous study showing lure response alone or target response alone represent poor markers of HE [27]. 

Anxiety is a relatively common feature of end-stage liver disease that occurs in 14%–45% of individuals with chronic liver disease [40], has a significant impact on health-related quality of life and functional capacity [41], and may vary with older age (>60 years) [42]. These results are the first to show an improvement in anxiety in the PP group who received combined treatment with synbiotics and BCAA (*p* = 0.035).

There are a variety of strategies utilised in the management of HE which predominantly focus on ammonia lowering strategies^1^, the most common being lactulose and rifaximin [1]. There have been reports of improved cognition, reduced risk of hospitalisation, and reduction in disease severity in patients with cirrhosis in response to supplementation with BCAAs [21] or the probiotic VSL#3 [18,43]. Reductions in endotoxemia, TNF, and improvement in dysbiosis demonstrated by beneficial changes in the stool microbial profile have also been reported in subjects who were taking the probiotic Lactobacillus GG [44]. These changes were not associated with a change in cognition [44]. 

This study could not demonstrate an improvement in levels of HE when the supplements were taken individually but, importantly, there was a significant improvement in cognition and, therefore, executive functioning when the treatments were combined. The positive results observed in the combined synbiotic and BCAA group suggest that synbiotics and BCAAs act synergistically to improve cognition and inhibition in patients with overt HE. Our results indicate that combined treatment with synbiotics and BCAA may be an additional and safe treatment option for the management of overt HE. A larger study is needed to confirm these results. 

### Study Limitations 

Due to the progressive nature of decompensated cirrhosis, 16% of participants who were originally recruited withdrew from the study due to worsening symptoms or voluntary abandonment, further limiting the data available for analysis. In addition, participants had MHE throughout the study which may be episodic, recurrent, or persistent [2]. Therefore, it is possible that participants exhibited fluctuating levels of HE at the time of testing. A larger study is suggested to investigate the effects of intervention with synbiotics and BCAAs on covert HE.

Analysis of the microbial diversity and richness of stool samples and assessment of intestinal epithelial permeability would have enhanced the results of this study but were not available at the time of the study design. Future studies in this area would benefit greatly by the addition of both microbial stool analysis and measures of intestinal epithelial permeability. Microbial analysis continues to evolve over time such that both a metagenomics and metabolomics approach would be ideal to identify not just the beneficial microbiota but also microbial products impacting HE.

As a result of the widespread adoption of rifaximin as a standard treatment, recruitment to this study was severely limited resulting in a small sample size. A larger study is needed to further explore the treatment effects which should include a rifaximin arm. 

## 5. Conclusions

This study is, to the best of our knowledge, the first to report the effects of combined oral supplementation with synbiotics and BCAAs in subjects with decompensated cirrhosis and MHE. The results demonstrate a positive treatment effect with the combined synbiotics and BCAAs. Although a larger study is recommended to investigate the effects of intervention with synbiotics and BCAAs on both covert and overt HE, these results suggest that oral supplementation with a combination of synbiotics and BCAAs may be an effective additional treatment for individuals with HE.

## Figures and Tables

**Table 1:** Baseline parameters of participants who returned for at least one follow-up assessment at 4 weeks and/or 8 weeks who were included in the intention-to-treat analysis. Data are presented as mean ± standard deviation unless otherwise stated.

	Placebo (*n* = 12)	Synbiotics (*n* = 12)	BCAA (*n* = 12)	Synbiotics + BCAA (*n* = 13)	Total (*n* = 49)
Gender (*n*)					
Male	11	11	9	11	42
Female	1	1	3	2	7
Age	54.1 ± 6.7	56.7 ± 7.5	5.7 ± 0.9	55.3 ± 4.4	55.8 ± 6.1
Primary Diagnosis (*n*)					
Viral	6	5	9	6	26
Alcohol	3	2	0	3	8
NASH	1	3	2	2	8
Cholestatic	2	0	0	1	3
Other	0	2	1	1	4
MELD	13.3 ± 2.9	13.2 ± 3.2	13.8 ± 2.9	14.4 ± 5.6	13.7 ± 3.8
CP score	9.2 ± 1.9	8.5 ± 0.9	9.2 ± 1.6	9.4 ± 2.4	9.1 ± 1.8
Albumin (g/L)	30.5.1 ± 5.3	34.6 ± 1.0	33.1 ± 7.2	33.3 ± 5.0	32.9 ± 5.6
Ammonia (µmol/L)	72.2 ± 29.5	84.0 ± 59.9	87.0.1 ± 37.9	80.8 ± 59.3	80.9 ± 47.7
Neutrophils (×10^9^ g/L)	3.0 ± 1.4	2.6 ± 1.3	3.2 ± 1.5	3.1 ± 1.4	3.0 ± 1.4
Lymphocyte (×10^9^ g/L)	1.1 ± 0.6	1.0 ± 0.4	1.0 ± 0.4	1.0 ± 0.5	1.0 ± 0.5
N:L ratio	3.0 ± 1.5	3.2 ± 1.5	4.1 ± 2.2	4.0 ± 02.4	3.6 ± 2.0
INR	1.5 ± 0.2	1.4 ± 0.2	1.5 ± 0.2	1.6 ± 0.4	1.5 ± 0.3
Ascites (*n*)					
None	4	2	0	4	10
Mild	3	2	6	4	15
Moderate	4	7	3	3	17
Severe	1	1	3	1	5
SGA (*n*)					
A	3	3	4	4	14
B	4	5	1	5	15
C	5	4	7	4	20
Daily Energy Intake (kcals/kg)	22.3 ± 6.4	23.4 ± 5.4	28.9 ± 6.5	26.7 ± 10.6	25.4 ± 7.7
Daily Protein (g/kg)	1.3 ± 0.6	1.2 ± 0.3	1.4 ± 0.4	1.3 ± 0.5	1.3 ± 0.4
Lactulose dose (mL/day)	72 ± 36	51 ± 40	67 ± 50	64 ± 56	64 ± 46
DASS-21					
Depression	12.8 ± 9.0	15.0 ± 10.3	12.2 ± 8.5	16.6 ± 9.6	14.2 ± 9.3
Anxiety	9.3 ± 7.7	10.2 ± 6.0	8.8 ± 7.9	12.0 ± 7.3	10.1 ± 7.2
Stress	15.2 ± 11.5	15.2 ± 10.3	15.2 ± 10.0	16.9 ± 11.9	15.6 ± 10.7
TMT (seconds)					
TMT A	49.9 ± 22.7	47.6 ± 15.6	47.4 ± 15.8	51.6 ± 17.7	49.2 ± 17.7
TMT B	119.3 ± 79.7	100.2 ± 66.8	123.4 ± 53.5	145.3 ± 69.3	122.5 ± 67.8
Inhibitory Control Test (ICT)					
Correct target response (*n*)	186.1 ±31.3	191.3 ± 21.1	196.2 ±17.4	184.4 ± 33.6	189.4 ± 26.4
Target accuracy *	0.88 ± 0.15	0.90 ± 0.10	0.93 ± 0.08	0.87 ± 0.16	0.89 ± 0.12
Incorrect lure response (*n*)	14.9 ± 10.9	13.9 ± 11.8	14.8 ± 12.1	14.0 ± 10.6	14.4 ± 11.0
Weighted lure response ^#^	24.6 ± 26.4	18.8 ± 18.2	18.3 ± 16.1	20.7 ± 15.8	20.6 ± 18.4

BCAA, branched chain amino acid; viral hepatitis includes hepatitis C and hepatitis B; NASH, non-alcoholic steatohepatitis; MELD, model for end-stage liver disease; CP, Child Pugh; N:L ratio, neutrophil-to-lymphocyte ratio; SGA, subjective global assessment of nutritional status; SGA A, well-nourished; SGA B, moderately malnourished; SGA C, severely malnourished; DASS-21, Depression-Anxiety-Stress short form; TMT, trail making test. * Target accuracy = correct target response ÷ total number targets; ^#^ weighted lure response = target accuracy ÷ incorrect lure response^2.^

**Table 2:** Trail Making Test (TMT) Performance.

Intention to Treat Analysis (ITT)	TMT-A (Seconds)	TMT-B (Seconds)
Placebo—baseline	49.93 ± 5.03	119.34 ± 17.97
Placebo—4 weeks	41.45 ± 5.03	107.82 ± 17.97
Placebo—8 weeks	41.29 ± 5.03	111.80 ± 17.97
Synbiotics—baseline	47.60 ±5.03	100.20 ± 17.97
Synbiotics—4 weeks	56.02 ± 5.17	123.44 ± 18.24
Synbiotics—8 weeks	49.53 ± 5.17	121.45 ± 18.24
BCAA—baseline	47.42 ± 5.03	123.44 ± 17.97
BCAA—4 weeks	43.30 ± 5.17	107.40 ± 18.24
BCCA—8 weeks	37.41 ± 5.33	110.08 ± 18.56
Synbiotics + BCAA—baseline	51.57 ± 4.83	145.3 ± 17.26
Synbiotics + BCAA—4 weeks	41.09 ± 4.83	104.72 ± 17.26
Synbiotics + BCAA—8 weeks	36.91 ± 4.83	94.56 ± 17.26 *
**Per protocol analysis (PP)**		
Placebo—baseline	45.46 ± 5.21	102.92 ± 17.65
Placebo—4 weeks	41.45 ± 5.03	107.82 ± 17.97
Placebo—8 weeks	41.29 ± 5.03	111.80 ± 17.97
Synbiotics—baseline	47.60 ±5.03	100.20 ± 17.97
Synbiotics—4 weeks	56.02 ± 5.17	123.44 ± 18.24
Synbiotics—8 weeks	49.53 ± 5.17	121.45 ± 18.24
BCAA—baseline	47.42 ± 5.03	123.44 ± 17.97
BCAA—4 weeks	43.30 ± 5.17	107.40 ± 18.24
BCCA—8 weeks	37.41 ± 5.33	110.08 ± 18.56
Synbiotics + BCAA—baseline	51.57 ± 4.83	145.3 ± 17.26
Synbiotics + BCAA—4 weeks	41.09 ± 4.83	104.72 ± 17.26
Synbiotics + BCAA—8 weeks	36.91 ± 4.83	94.56 ± 17.26 ^#^

Linear mixed models analysis: Data presented as estimated means ± standard error for fixed effects from linear mixed models. * ITT analysis of TMT B results demonstrated a significant reduction in time taken to complete TMT B in the combined synbiotics and BCAA treatment group at 8 weeks versus baseline in the placebo group (*p* = 0.018). ^#^ PP analysis of TMT B results demonstrated a significant reduction in time taken to complete TMT B in the combined synbiotics and BCAA treatment group at 8 weeks versus baseline in the placebo group (*p* = 0.017).

**Table 3:** Inhibitory Control Test (ICT) Responses.

Intention to Treat Analysis (ITT)	Correct Target Responses	Target Accuracy	Incorrect Lure Responses	Weighted Lures
Placebo—Baseline	186.08 ± 7.33	0.88 ± 0.04	14.92 ± 3.00	24.55 ± 4.33
Placebo—4 weeks	201.50 ± 7.33	0.95 ± 0.04	10.67 ± 3.00	12.47 ± 4.33
Placebo—8 weeks	197.08 ± 7.33	0.93 ± 0.04	9.42 ± 3.00	12.71 ± 4.33
Synbiotics—baseline	191.33 ± 7.33	0.90 ± 0.04	13.92 ± 3.00	18.81 ± 4.33
Synbiotics—4 weeks	184.27 ± 7.89	0.79 ± 0.04	11.12 ± 3.05	16.53 ± 4.45 *
Synbiotics—8 weeks	197.66 ± 7.59	0.93 ± 0.04	12.36 ± 3.05	15.27 ± 4.39 **
BCAA—baseline	197.17 ± 7.33	0.93 ± 0.04	14.75 ± 3.00	18.31 ± 4.33
BCAA—4 weeks	199.34 ± 7.59	0.94 ± 0.04	13.42 ± 3.05	15.05 ±4.39 *
BCCA—8 weeks	198.04 ± 7.89	0.93 ± 0.04	14.23 ± 3.11	19.97 ± 4.45 **
Synbiotics + BCAA—baseline	184.38 ± 7.05	0.87 ± 0.04	14.00 ± 2.88	20.70 ± 4.16
Synbiotics + BCAA—4 weeks	183.85 ± 7.05	0.87 ± 0.04	10.77 ± 2.88	15.80 ± 4.16 **
**Per protocol analysis (PP)**				
Placebo—baseline	191.64 ± 7.70	0.90 ±0.04	13.82 ± 3.10	19.72 ± 4.24
Placebo—4 weeks	203.85 ± 8.03	0.96 ± 0.04	8.46 ± 3.16	8.93 ± 4.30 ^##^
Placebo—8 weeks	202.20 ± 8.41	0.95 ± 0.05	7.21 ± 3.23	9.50 ± 4.37 ^##^
Synbiotics—baseline	189.80 ± 8.07	0.90 ± 0.04	14.80 ± 3.25	20.47 ± 4.45
Synbiotics—4 weeks	179.01 ± 9.48	0.74 ± 0.05	10.60 ± 3.41	16.24 ± 4.73
Synbiotics—8 weeks	202.14 ± 8.93	0.94 ± 0.05	10.90 ± 3.41	13.38 ± 4.62 ^#^
BCAA—baseline	191.67 ± 8.51	0.90 ± 0.05	16.67 ± 3.43	21.35 ± 4.69
BCAA—4 weeks	202.03 ± 9.52	0.95 ± 0.05	15.90 ± 3.61	17.82 ± 4.88
BCCA—8 weeks	192.56 ± 9.52	0.91 ± 0.05	15.92 ± 3.61	19.50 ± 4.88 ^#^
Synbiotics + BCAA—baseline	184.38 ± 7.08	0.87 ± 0.04	14.00 ± 2.85	20.70 ± 3.90
Synbiotics + BCAA—4 weeks	182.10 ± 7.96	0.86 ± 0.04	12.39 ± 3.01	18.32 ± 4.06
Synbiotics + BCAA—8 weeks	199.16 ± 7.33	0.94 ± 0.04	8.21 ± 2.90 *	9.38 ± 3.95 ^#^

Results expressed as mean ± SE; Weighted lures = incorrect lure responses ÷ target accuracy^2^; BCAA, branched chain amino acids. ITT analysis: * *p* < 0.05: there was a significant reduction in weighted lures at 4 weeks for the synbiotic and BCAA alone and combined synbiotic BCAA groups compared to baseline placebo. ** *p* < 0.05: there was a significant change from baseline in weighted lures at 8 weeks for the synbiotics and BCAA alone groups compared to the changes in the placebo group. *** *p* < 0.05: there was a significant reduction in in weighted lures in the combined synbiotic and BCAA treated group at 8 weeks compared to placebo. PP analysis: ^#^ *p* < 0.05: At 8 weeks there was a significant reduction in the weighted lures for the synbiotics, BCAA alone groups, and the combined synbiotic and BCAA group compared to the baseline placebo group. ^##^ *p* < 0.05: There was a significant reduction in weighted lures for placebo at week 4 and week 8 compared to baseline.

**Table 4:** Depression, Anxiety and Stress Scale - Short Form Responses.

Intention to Treat Analysis (ITT)	Depression	Anxiety	Stress
Placebo—Baseline	12.83 ± 2.57	9.33 ± 1.99	15.17 ± 3.03
Placebo—4 weeks	12.30 ± 2.63	10.06 ± 2.03	11.09 ± 3.07
Placebo—8 weeks	10.17 ± 2.57	10.50 ± 1.99	13.67 ± 3.03
Synbiotics—baseline	15.00 ± 2.57	10.17 ± 1.99	15.00 ± 3.03
Synbiotics—4 weeks	12.69 ± 2.63	10.24 ± 2.03	12.22 ± 3.07
Synbiotics—8 weeks	12.57 ± 2.70	11.65 ± 2.06	18.17 ± 3.12
BCAA—baseline	12.17 ± 2.57	8.83 ± 1.99	15.17 ± 3.03
BCAA—4 weeks	13.49 ± 2.63	9.95 ± 2.03	15.96 ± 3.07
BCCA—8 weeks	12.19 ± 2.70	9.19 ± 2.06	14.12 ± 3.12
Synbiotics + BCAA—baseline	16.62 ± 2.47	12.00 ± 1.92	16.92 ± 2.91
Synbiotics + BCAA—4 weeks	14.77 ± 2.47	10.31 ± 1.92	14.15 ± 2.90
Synbiotics + BCAA—8 weeks	13.38 ± 2.47	9.54 ± 1.92	14.15 ± 2.90
**Per Protocol analysis (PP)**			
Placebo—Baseline	12.91 ± 2.70	9.64 ±2.07	16.55 ± 3.22
Placebo—4 weeks	12.99 ± 2.77	10.46 ±2.11	12.04 ± 3.26
Placebo—8 weeks	12.15 ± 2.86	11.28 ± 2.15	16.14 ± 3.32
Synbiotics—baseline	16.40 ± 2.83	11.00 ± 2.17	16.40 ± 3.37
Synbiotics—4 weeks	12.64 ± 3.02	10.61 ± 2.26	11.72 ± 3.50
Synbiotics—8 weeks	15.01 ± 3.14	11.77 ± 2.32	21.31 ± 3.58
BCAA—baseline	14.44 ± 2.99	8.44 ± 2.29	17.11 ± 3.56
BCAA—4 weeks	15.26 ± 3.21	8.06 ± 2.40	19.89 ± 3.70
BCCA—8 weeks	12.06 ± 3.21	7.81 ± 2.40	16.03 ± 3.70
Synbiotics + BCAA—baseline	16.62 ± 2.49	12.00 ± 1.91	16.92 ± 2.96
Synbiotics + BCAA—4 weeks	13.97 ± 2.67	8.95 ± 2.00	14.23 ± 3.08
Synbiotics + BCAA—8 weeks	12.79 ± 2.54	9.44 ± 1.93 *	14.20 ± 2.99

Results expressed as estimated mean ± standard error for fixed effects from linear mixed models. * *p* = 0.035: At 8 weeks there was a significant reduction in anxiety observed in the combined synbiotics and BCAA group compared to placebo.
